# Determining the validity of non-invasive spot-check hemoglobin co-oximetry testing to detect anemia in postpartum women at a tertiary care centre, a prospective cohort study

**DOI:** 10.1186/s12884-023-05783-3

**Published:** 2023-06-29

**Authors:** Kienna Mills, Julie M. Vermeer, Warren E. Berry, Erwin Karreman, Christine D. Lett

**Affiliations:** 1 Health Science Building, University of Saskatchewan, 107 Wiggins Rd, Saskatoon, SK S7N 5E5 Canada; 2 Department of Obstetrics and Gynecology, University of Saskatchewan, Reginal General Hospital, 1440 14 Ave, ReginaRegina, Saskatchewan S4P 0W5 Canada; 3 Saskatchewan Health Authority, 2180 23rd Avenue, Regina, SK S4S 0A5 Canada

**Keywords:** Postpartum, Anemia, Non-invasive hemoglobin measurement, Spot-check hemoglobin co-oximetry, Patient blood management

## Abstract

**Background:**

Spot-check hemoglobin co-oximetry analyzers measure hemoglobin transcutaneously and offer the benefit of a hemoglobin measurement without phlebotomy. The objective of this study was to determine the validity of non-invasive spot-check hemoglobin co-oximetry testing for the detection of postpartum anemia (hemoglobin < 10 g/dL).

**Methods:**

Five hundred eighty-four women aged 18 and over were recruited on postpartum day one following a singleton delivery. Two non-invasive spot-check hemoglobin co-oximetry monitors, Masimo Pronto Pulse CO-Oximeter (Pronto) and Masimo Rad-67 Pulse CO-Oximeter (Rad-67), were evaluated and compared to the postpartum phlebotomy hemoglobin value.

**Results:**

Of 584 participants, 31% (181) had postpartum anemia by phlebotomy hemoglobin measurement. Bland–Altman plots determined a bias of + 2.4 (± 1.2) g/dL with the Pronto and + 2.2 (± 1.1) g/dL with the Rad-67. Low sensitivity was observed: 15% for the Pronto and 16% for the Rad-67. Adjusting for the fixed bias, the Pronto demonstrated a sensitivity of 68% and specificity of 84%, while the Rad-67 demonstrated a sensitivity of 78% and specificity of 88%.

**Conclusion:**

A consistent overestimation of hemoglobin by the non-invasive spot-check hemoglobin co-oximetry monitors compared to phlebotomy hemoglobin result was observed. Even after adjusting for the fixed bias, the sensitivity for detecting postpartum anemia was low. Detection of postpartum anemia should not be based on these devices alone.

## Background

38% of women worldwide are anemic during pregnancy [1]. During a vaginal or caesarean delivery, it is common for women to have 500 mL or 1000 mL of blood loss, respectively. Postpartum anemia is defined as hemoglobin < 10.0 g/dL [2] and iis present in 22% of women in developed countries [3]. Postpartum anemia is associated with fatigue, breathlessness, palpitations, impaired lactation, reduced cognitive abilities, emotional instability, depression, and compromised mother–child bonding [1]. Given the prevalence of postpartum anemia and its associated morbidity, our institution routinely measures hemoglobin by complete blood count (CBC) on postpartum day one to detect and facilitate the treatment of anemia.

Several non-invasive hemoglobin monitors have been developed. The Masimo Pronto Pulse CO-Oximeter (Pronto Pulse) and Masimo Rad-67 Pulse CO-Oximeter (Rad-67) use infrared light to measure hemoglobin based on its specific absorption characteristics. The subsequent spectrophotometric analysis provides an immediate measurement of transcutaneous hemoglobin, referred to as spot-check hemoglobin co-oximetry (SpHb). These CO-Oximeters have been marketed to have an accuracy of ± 1.0 g/dL [4]. In a systematic review of CO-Oximeters, Hiscock et al. [4] reported a bias of + 0.18 g/dL for the Pronto device and -0.11 g/dL for the Rad-67.

Multiple studies have looked at spot-check hemoglobin co-oximetry devices in different populations [4–15]. Most of these studies reviewed patients with normal hemoglobin values. Joseph et al. [12] conducted a study with a significant cohort of anemic patients. They assessed the CO-Oximeter in 525 trauma patients, including 173 patients with hemoglobin ≤ 8.0 g/dL. They stratified patients into two groups based on the laboratory hemoglobin result and the SpHb using a cut-off of 8.0 g/dL. They found a sensitivity of 95.4% and specificity of 63.8% using the device to predict anemia below 8.0 g/dL.

Morey et al. [16] contend that evaluating hemoglobin monitors in patients with normal hemoglobin is insufficient to make decisions regarding anemic patient management. They propose that the device be precise in the range that impacts patient care decisions, 6.0 g/dL to 10.0 g/dL. If hemoglobin is < 6.0 g/dL, there will be clinical indicators that warrant action, such as transfusion. If hemoglobin is > 10.0 g/dL, the patient would not need treatment. Significant errors would occur if the device read > 10.0 g/dL when the hemoglobin was < 6.0 g/dL and vice versa. Morey et al. [16] recommend using a hemoglobin error grid to determine the clinical usefulness of CO-Oximeters. Furthermore, Applegate et al. [7] developed a hemoglobin error grid based on the work of Morey et al. when comparing three methods of intraoperative hemoglobin trend assessment (SpHb, arterial blood gas co-oximetry, and point of care analyzers to traditional CBC). While this development showed utility, it had the limitation in that only 10% of included samples had hemoglobin < 8.0 g/dL making it challenging to assess clinical utility at low hemoglobin levels. These findings support using an exclusion zone model for hemoglobin monitors which will vary based on different analyzers.

The CO-Oximeters have not been well studied in postpartum women, particularly women with postpartum anemia. Four studies have examined non-invasive hemoglobin monitors in pregnant and postpartum women [4, 6, 7, 14]. Two of these studies looked at the Rad-67 CO-Oximeter [6, 7], and two looked at the Pronto Pulse CO-Oximeter [4, 14]. These did not specifically look at anemia. The primary objective of our study was to evaluate the validity of the CO-Oximeters in detecting postpartum anemia compared to invasive laboratory testing. The secondary objectives were to determine the device reliability, sensitivity, specificity, and predictive value of the CO-Oximeters to identify postpartum anemia, and to evaluate if the mode of delivery impacts device accuracy. This is the first CO-Oximeter comparison study to determine the accuracy of postpartum anemia detection using a one-time spot check.

## Methods

This was a prospective evaluation of two spot-check hemoglobin CO-Oximeter devices conducted over two time periods. The first data collection included an analysis of the Masimo Pronto Pulse CO-Oximeter alone. The second data collection included analysis of both the Masimo Pronto Pulse CO-Oximeter and Masimo Rad-67 Co-Oximeter. Data collected between the two study arms were further combined and analyzed.

Approval for both studies was obtained from Saskatchewan Health Authority Research Ethics Board. Written consent was obtained from participants. Data collected included patient demographics (age, gravidity, parity, ethnicity, body mass index), gestational age at delivery, mode of delivery, time of delivery, physician estimation of postpartum blood loss, time of CBC collection, phlebotomy hemoglobin result, CO-Oximeter testing time, and CO-Oximeter results.

Using the Pronto device, a single investigator recruited a convenience sample of women on postpartum day one following singleton deliveries at a tertiary care center from July to September 2019. A second investigator recruited a convenience sample of women on postpartum day one following singleton deliveries at the same tertiary care centre from May to September 2021 using both the Pronto and Rad-67 devices.

During initial data collection, three consecutive measurements of SpHb using the Pronto device were collected (*n* = 283). Rad-67 samples were collected in triplicate for the first twenty measurements. Once high device reliability was determined, a single SpHb measurement was taken on each device.

The SpHb device reports heart rate, oxygen saturation, perfusion index, and total hemoglobin using a measurement probe applied to the finger in a similar fashion to a standard pulse oximeter. SpHb measurements were collected following Massimo’s directions for use, applying the probe to the index finger of the patient’s non-dominant hand for the Pronto device and applying the probe to the non-dominant fifth finger for the Rad-67. It took approximately five minutes to collect three measurements from each patient.

**Fig. 1:**
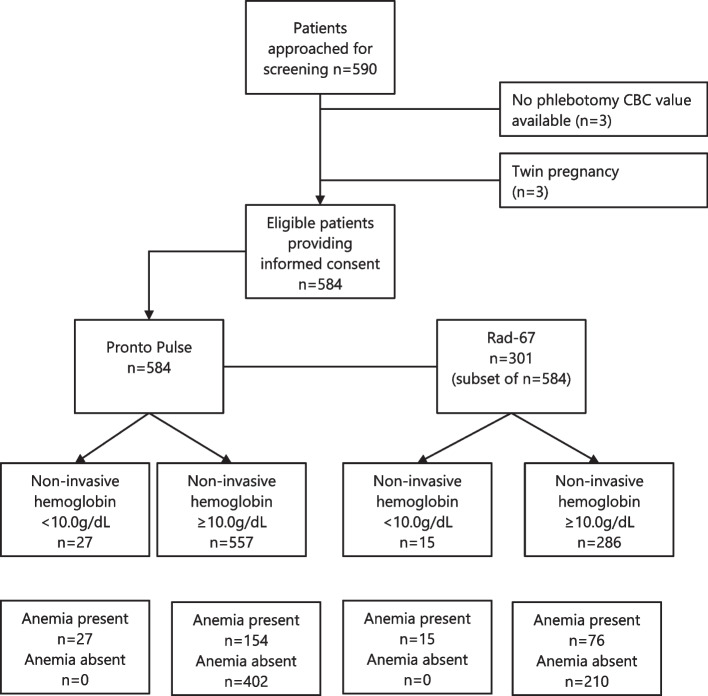
STAndards for the Reporting of Diagnostic accuracy studies (STARD) flow diagram

Serum hemoglobin was determined using the accredited hospital lab’s calibrated Beckman Coulter UniCel DXH 800 analyzer, and these results were collected from the patient chart. It was not possible to arrange serum hemoglobin and SpHb collection simultaneously. This study adheres to Standards for Accurate Reporting of Diagnostic Tests (STARD) guidelines (Fig. 1).

Intraclass correlation coefficient (ICC) was used to assess device reliability. Student t-tests were used to determine if there was a significant difference in device accuracy based on the mode of delivery. Bland–Altman analysis assessed the agreement between invasive and non-invasive hemoglobin results. Hemoglobin error grids were created for both devices modelled after the hemoglobin error grids created by Morey et al. [14]. The data were grouped by serum hemoglobin ≥ 10.0 g/dL and < 10.0 g/dL to determine the predictive value of the device to identify postpartum anemia. Data were recorded in Microsoft Excel, and analysis was carried out using R Core Team 2018. Assuming an ICC of 0.9 and a research hypothesis of an ICC of 0.95 (alpha of 0.05 and power of 0.80), a minimum of 50 patients were required for each of the following subgroups: normal hemoglobin (≥ 10.0 g/dL), anemia (< 10.0 g/dL), vaginal delivery, and caesarean delivery.

## Results

Initial data were collected on 283 postpartum women using Pronto CO-Oximeter alone. Subsequent data were collected on 301 postpartum women using both the Rad-67 and Pronto CO-Oximeters. Data were pooled for the Pronto device (*n* = 584). Patient demographics are summarized in Table 1.

**Table 1:** Patient demographics

	Pronto* (*n* = 584)	Rad-67^+^ (*n* = 301)
Age, years (SD)	30.38 (4.85)	30.64 (4.96)
Gravidity (SD)	2.40 (1.60)	2.43 (1.75)
Parity (SD)	1.48 (1.30)	1.91 (1.17)
Body mass index (SD)	30.93 (7.11)	29.96 (7.77)
Vaginal delivery, n (%)		
- Total - Spontaneous - Forcep assisted - Vacuum assisted	389 (66.6) 320 (82.3) 15 (3.9) 54 (13.9)	204 (67.8) 167 (81.9) 8 (3.9) 29 (14.2)
Caesarean Section, n (%)	195 (33.4)	97 (32.2)
Postpartum anemia (Hemoglobin < 10.0 g/dL), n (%)	182 (31.2)	91 (30.2)
Reported postpartum hemorrhage, n (%)	79 (13.5)	51 (16.9)
- Patients with postpartum hemorrhage and postpartum anemia found by CO-Oximeter	54 (68.4)	31 (60.8)
Time between CBC and SpHb (SD)	120 (100)	116 (97)

Characteristics of patients who participated in the study. Results are median [25th to 75th percentile] except for mode of delivery (vaginal, caesarean), postpartum anemia, and hemorrhage which are number (%)

In the Pronto group of 584 women, 67% had a vaginal delivery, 31% had postpartum anemia, and 14% had a postpartum hemorrhage. Serum hemoglobin ranged from 5.4 to 14.5 g/dL. The mean time from collection of CBC and hemoglobin measurement with the CO-Oximeter was 120 min. For the Rad-67 device (*n* = 301), 68% had a vaginal delivery, 30% had postpartum anemia, and 17% had a postpartum hemorrhage. Serum hemoglobin ranged from 4.8 to 14.5 g/dL. The mean time from collection of CBC and hemoglobin measurement with the CO-Oximeter was 116 min.

**Fig. 2:**
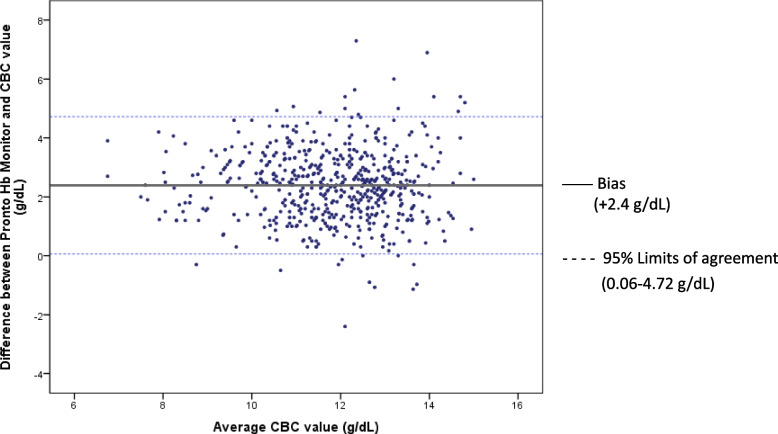
Bland Altman analysis comparing average of Pronto CO-Oximeter spot-check hemoglobin co-oximetry (SpHb) and laboratory hemoglobin result. Bland–Altman analysis comparing phlebotomy hemoglobin to Pronto spot-check hemoglobin co-oximetry (SpHb); Outer margin lines indicate 95% limits of agreement

**Fig. 3:**
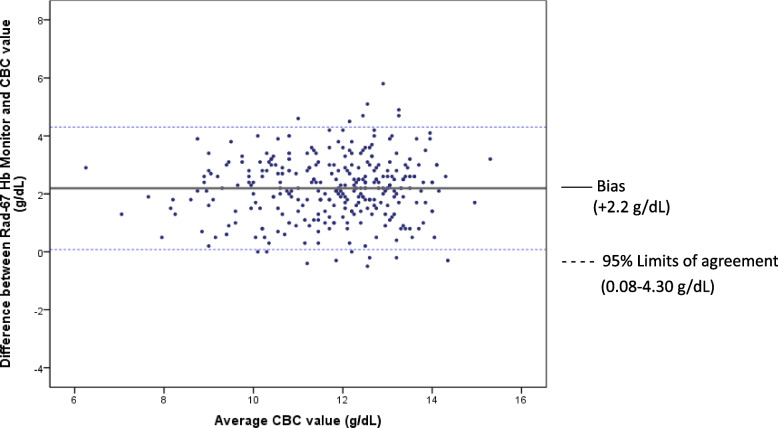
Bland Altman analysis comparing average of Rad-67 CO-Oximeter spot-check hemoglobin co-oximetry (SpHb) and laboratory hemoglobin result. Bland–Altman analysis comparing phlebotomy hemoglobin to Rad-67 spot-check hemoglobin co-oximetry (SpHb); Outer margin lines indicate 95% limits of agreement

The average SpHb value was compared to the serum hemoglobin results using Bland–Altman analysis. A bias of + 2.4 g/dL (SD = 1.2 g/dL) was demonstrated for the Pronto (Fig. 2). The limits of agreement (LOA) are the 95% confidence interval, 0.06–4.72 g/dL, with an absolute width of 4.66 g/dL. Bland–Altman analysis for the Rad-67 showed a bias of + 2.2 g/dL (SD = 1.1 g/dL) (Fig. 3). The LOA was 0.08–4.30 g/dL with an absolute width of 4.22 g/dL.

**Fig. 4:**
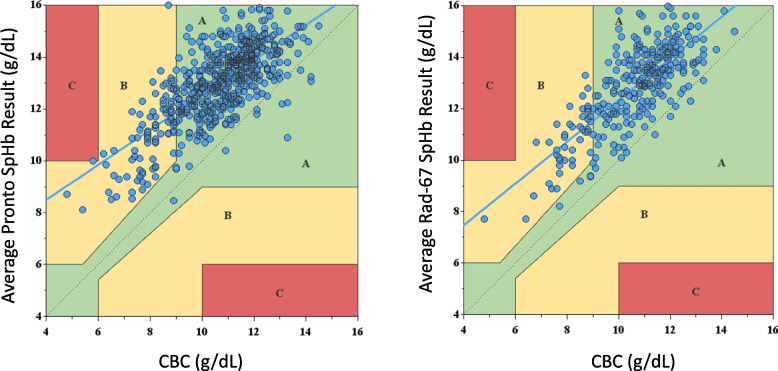
Hemoglobin Error Grid for Pronto and Rad-67 CO-Oximeter versus laboratory hemoglobin, adjusted for fixed bias*. Clinical acceptability plot of accuracy comparing hemoglobin determined by phlebotomy to spot-check hemoglobin co-oximetry (SpHb). *Fixed bias for Pronto + 2.4 g/dL, for Rad-67 + 2.2 g/dL. –– Line of Unity: Ideally all data points fall along this line. Zone A (Green) indicates SpHb results within a clinically acceptable range. Zone B (Yellow) indicates SpHb results that could represent a clinically significant error. Zone C (Red) indicates a potentially dangerous error in results from SpHb. See text for the significance of each zone

Hemoglobin error grids for both devices were developed (Fig. 4), with the CBC result plotted against the average SpHb for each patient. The SpHb was modified for the fixed biases found in this study. The dispersion around the line of unity represents the imprecision of the non-invasive hemoglobin measurement. Zone B should contain < 5% of data points, and no data points should fall in Zone C. The hemoglobin error grid for the Pronto shows 16.8% of data points fell in Zone B with one measurement in Zone C, while the Rad-67 had 15.6% data points in Zone B with none in Zone C.

Intraclass correlation (ICC) was calculated using the three SpHb values for each patient in the first set of data collection for the Pronto (*n* = 283) and the first 20 patients for the Rad-67. The ICC was 0.948 (95% confidence interval 0.923 – 0.963) for the Pronto and 0.948 (95% confidence interval 0.925 – 0.964) for Rad-67, demonstrating excellent device reliability. Once this reliable ICC was established, one measurement was taken to accelerate data collection.

When it was determined that a single instrument gave consistently reliable results, we compared those results to the gold standard of phlebotomy. The Pronto had an ICC of 0.33, and the Rad-67 device had an ICC of 0.4 compared to lab hemoglobin. When the two devices were compared, the ICC was 0.74.

The difference between the average SpHb value and hemoglobin result was compared with vaginal and caesarean delivery using a student T-test. There was no significant difference (*p* > 0.05) between the two modes of delivery.

Hemoglobin results were grouped based on the presence of postpartum anemia based on phlebotomy hemoglobin < 10 g/dL to calculate sensitivity, specificity, and predictive value. (Table 2). The sensitivity of the Pronto device was 14.9%, and the specificity was 100%. When this was adjusted for the fixed bias of + 2.4 g/dL found in this study, using a SpHb cut-off for the Pronto device of 12.4 g/dL, a sensitivity of 67.9% and a specificity of 83.6% was demonstrated. The positive predictive value was 65.1%, and the negative predictive value of 85.3% when adjusted for fixed bias (Table 3).

**Table 2:** Pronto (*n* = 584) CO-Oximeter predictive value for postpartum anemia

		Serum Hemoglobin		
		< 10.0 g/dL	≥ 10.0 g/dL	
SpHb	< 10.0 g/dL	27	0	PPV 100%
	≥ 10.0 g/dL	154	402	NPV 72.3%
		Sensitivity 14.9%	Specificity 100%	

Calculation of sensitivity, specificity, positive and negative predictive value for each device

*SpHb* Spot-check hemoglobin co-oximetry

*PPV* Positive predictive value

*NPV* Negative predictive value

**Table 3:** Pronto (*n* = 584) CO-Oximeter predictive value for postpartum anemia, SpHb adjusted for fixed bias

		Serum Hemoglobin		
		< 10.0 g/dL	≥ 10.0 g/dL	
SpHb	< 12.3 g/dL	123	66	PPV 65.1%
	≥ 12.3 g/dL	58	336	NPV 85.3%
		Sensitivity 68.0%	Specificity 83.6%	

Calculation of sensitivity, specificity, positive and negative predictive value for each device

*SpHb* Spot-check hemoglobin co-oximetry

*PPV* Positive predictive value

*NPV* Negative predictive value

The Rad-67 had a sensitivity of 16.4% and specificity of 100% to detect postpartum anemia, with a positive predictive value of 100% and a negative predictive value of 73.4% (Table 4). When this was adjusted for the fixed bias of + 2.2 g/dL, the sensitivity was 78.0%, and the specificity was 88.1%. The positive predictive value was 74.0%, and the negative predictive value was 90.2% when adjusted for fixed bias (Table 5).

**Table 4:** Rad-67 (*n* = 301) CO-Oximeter predictive value for postpartum anemia Rad-67

		Serum Hemoglobin		
		< 10.0 g/dL	≥ 10.0 g/dL	
SpHb	< 10.0 g/dL	15	0	PPV 100%
	≥ 10.0 g/dL	76	210	NPV 73.4%
		Sensitivity 16.4%	Specificity 100%	

Calculation of sensitivity, specificity, positive and negative predictive value for each device

*SpHb* Spot-check hemoglobin co-oximetry

*PPV* Positive predictive value

*NPV* Negative predictive value

**Table 5:** Rad-67 (*n* = 301) CO-Oximeter predictive value for postpartum anemia, SpHb adjusted for fixed bias + 2.2 g/dL

		Serum Hemoglobin		
		< 10.0 g/dL	≥ 10.0 g/dL	
SpHb	< 12.2 g/dL	71	25	PPV 74.0%
	≥ 12.2 g/dL	20	185	NPV 90.2%
		Sensitivity 78.0%	Specificity 88.1%	

Calculation of sensitivity, specificity, positive and negative predictive value for each device

*SpHb* Spot-check hemoglobin co-oximetry

*PPV* Positive predictive value

*NPV* Negative predictive value

## Discussion

Multiple studies have compared spot-check hemoglobin co-oximetry to laboratory hemoglobin values in a variety of populations, including patients presenting to outpatient labs, healthy volunteers, potential blood donors, emergency room patients, trauma patients, surgical patients, and pregnant and postpartum women [5–7, 9–14, 17, 18]. This study looked at women postpartum day one following vaginal or caesarean delivery. This study has the largest cohort of postpartum women and includes the most women with postpartum anemia to date.

The bias between CO-Oximeter (Pronto + 2.4 g/dL and Rad-67 + 2.2 g/dL) and CBC was much higher than the reported manufacturer accuracy of ± 1.0 g/dL and the systematic review reported bias of + 0.18 g/dL for the Pronto device and -0.11 g/dL for the Rad-67 [4]. After analysis of initial Pronto data (*n* = 283), it was unclear if the bias noted was device, user, or situation specific. Therefore, data collection using two CO-Oximeters aimed to address the possible limitation of a poorly calibrated single device. The devices showed an ICC of 0.74, suggesting moderate reliability between the devices. However, compared to the phlebotomy hemoglobin, the reliability was low for each device (ICC < 0.5). Given the persistent biases in both devices, poor device calibration is less likely.

Factors that may impact device bias include: the sex of patients studied, with improved performance in male patients [19]; sex differences in perfusion index [19] with lower perfusion index in women [20]; and poorer device performance when hemoglobin is outside of the optimal level of device calibration [20]. The larger-than-expected biases observed in our study may be due to the female population with many anemic patients.

Hemoglobin error grids (Fig. 4) were modelled after the hemoglobin error grids created by Morey et al. [16] and modified for the fixed biases found in this study as Applegate et al. concluded that the error grid would need to be modified for each device [7]. The CBC result and average SpHb were plotted as ordered pairs. If all points fell on the line of unity, it would indicate a perfect correlation between CBC and SpHb once adjusted for the fixed bias. The dispersion around the unity line represents the SpHb’s imprecision. Furthermore, the hemoglobin error grids demonstrate the SpHb measurements could lead to clinical error. The error grid is divided into three zones. Ideally Zone A contains 95% of hemoglobin measurements. It includes the region < 6.0 g/dL, as there should be clinical indicators for transfusion at this level. Zone A also includes the region > 10.0 g/dL; above this value, patients are beyond the point where transfusion should be considered. The critical portion of Zone A is the isthmus where hemoglobin results may impact clinical decision-making (for example, prescribing red blood cell transfusion or intravenous iron). As such, the CO-Oximeter and CBC result must agree most closely in this region. Zone B represents a significant discrepancy between SpHb and CBC results. It should contain < 5% of data points. Zone C represents the critical error zone where an incorrect CO-Oximeter result could cause patient harm (inappropriate transfusion with exposure to transfusion complications or withholding a transfusion where it is indicated). There should be no points located in Zone C. Figure 4 demonstrates that these devices do not meet these specifications as 16.8% and 15.6% of results fell in Zone B for the Pronto and Rad-67, respectively, with one measurement in Zone C for the Pronto.

Good screening tests have high sensitivity. Tables 2 and 4 demonstrate that with a SpHb < 10.0 g/dL, the sensitivity for detecting postpartum anemia in this study was very low (Pronto 15%, Rad-67 16%). It was hypothesized that adjusting for fixed bias, as determined by Bland–Altman plots, would improve the sensitivity of the CO-Oximeter. Tables 3 and 5 demonstrate that adjustment did increase the sensitivity to 68% for the Pronto and 78% for the Rad-67. Even with adjustment for bias, these devices still misclassify many anemic patients.

A recent study in anemic ICU patients concluded that non-invasive spot-check hemoglobin co-oximetry was not sufficiently accurate for clinical utility in their patient population [20, 21]. Given the results of our study and the potential for clinical error as demonstrated on the hemoglobin error grid, we also conclude that SpHb is not sufficiently accurate for detecting anemia in postpartum patients.

The main strength of this study is the large number of anemic patients; 176 women with hemoglobin < 10.0 g/dL, 102 of whom had hemoglobin < 9.0 g/dL. Other studies using the Pronto and Rad-67 devices evaluated patients with relatively normal hemoglobin values [8] and have been criticized for extrapolating to anemic patients [12].

Limitations of this study include not recording the perfusion index, and the time gap between CBC collection and SpHb testing, which could be significant in the case of a delayed postpartum hemorrhage between measurements. Ideally, all SpHb measurements would be collected in triplicate; however, this proved to be time and cost prohibitive. Once the individual device ICC was determined to be excellent, single measurements were obtained.

Future research exploring how to decrease unnecessary phlebotomy in women at low risk of postpartum anemia is needed to facilitate patient blood management in postpartum women. Creating and validating a screening tool based on historical risk factors for postpartum anemia, such as those proposed by Bergmann et al. [2], combined with CO-Oximeter device assessment to stratify women into low, medium, and high risk for postpartum anemia could allow women at low risk of postpartum anemia to forgo phlebotomy hemoglobin testing.

## Conclusions

The non-invasive spot-check hemoglobin co-oximetry monitors consistently overestimated the hemoglobin value compared to phlebotomy hemoglobin. Adjusting for the fixed bias did not adequately improve the sensitivity for detecting postpartum anemia. The CO-Oximeters in our study had a higher positive bias but narrower limits of agreement than previously described [4]. Due to the high variance, individual patients may be misclassified as normal when anemic. While it would be ideal to use a non-invasive screening tool to assess for postpartum anemia, the CO-Oximeters evaluated need further refinement before implementation in the postpartum setting. Due to the relatively low cost and ease of access to phlebotomy, a CBC remains the gold standard for detecting postpartum anemia.

## Data Availability

The datasets used and analyzed during the current study are available from the corresponding author upon reasonable request.

## Abbreviations

Rad-67: Rad-67 CO-Oximeter
Pronto Pulse: Masimo Pronto Pulse CO-Oximeter
SpHb: Spot-check hemoglobin co-oximetry
CBC: Complete blood count
LOA: Limits of agreement
ICC: Intraclass correlation coefficient
SD: Standard deviation
PPV: Positive predictive value
NPV: Negative predictive value
